# Comprehensive Evaluation of the Toxicity and Biosafety of Plasma Polymerized Nanoparticles

**DOI:** 10.3390/nano11051176

**Published:** 2021-04-29

**Authors:** Praveesuda L. Michael, Yuen Ting Lam, Juichien Hung, Richard P. Tan, Miguel Santos, Steven G. Wise

**Affiliations:** 1 Faculty of Health and Medicine, School of Medical Sciences, University of Sydney, Sydney, NSW 2006, Australia; praveesuda.michael@sydney.edu.au (P.L.M.); yuen.lam@sydney.edu.au (Y.T.L.); chien.hung@sydney.edu.au (J.H.); richard.tan@sydney.edu.au (R.P.T.); 2 Charles Perkins Centre, University of Sydney, Sydney, NSW 2006, Australia; 3 The University of Sydney Nano Institute (Sydney Nano), University of Sydney, Sydney, NSW 2006, Australia

**Keywords:** plasma polymerized nanoparticle, dusty plasmas, cellular cytotoxicity, nanotoxicity, biocompatibility

## Abstract

The rapid growth of nanoparticle-based therapeutics has underpinned significant developments in nanomedicine, which aim to overcome the limitations imposed by conventional therapies. Establishing the safety of new nanoparticle formulations is the first important step on the pathway to clinical translation. We have recently shown that plasma-polymerized nanoparticles (PPNs) are highly efficient nanocarriers and a viable, cost-effective alternative to conventional chemically synthesized nanoparticles. Here, we present the first comprehensive toxicity and biosafety study of PPNs using both established in vitro cell models and in vivo models. Overall, we show that PPNs were extremely well tolerated by all the cell types tested, significantly outperforming commercially available lipid-based nanoparticles (lipofectamine) used at the manufacturer’s recommended dosage. Supporting the in vitro data, the systemic toxicity of PPNs was negligible in BALB/c mice following acute and repeated tail-vein intravenous injections. PPNs were remarkably well tolerated in mice without any evidence of behavioral changes, weight loss, significant changes to the hematological profile, or signs of histological damage in tissues. PPNs were tolerated at extremely high doses without animal mortality observed at 6000 mg/kg and 48,000 mg/kg for acute and repeated-injection regimens, respectively. Our findings demonstrate the safety of PPNs in biological systems, adding to their future potential in biomedical applications.

## 1. Introduction

The number of nanocarriers has vastly increased in recent decades, with potential implementation across a range of biomedical fields, including targeted drug delivery [1], device-based therapy [2], medical imaging [3], regenerative medicine, and tissue engineering [4]. The unique features of nanoparticles, including their small size, high surface-to-volume ratio, and design versatility, allows for a wide selection of payloads, compositions, and targeting moieties [5,6]. These characteristics enable nanocarriers to overcome the pharmacokinetic limitations of conventional drug formulations by breaking through biological barriers and prolonging the accumulation and retention at the targeted site, thereby increasing the efficacy of the drugs [7]. For instance, the distribution of doxorubicin (Dox) as a free drug is reported to be less than 5 min in circulation. Meanwhile, self-assembled lipid–polymer hybrid nanoparticles of Dox prolong the circulation of the drug and increase its accumulation at the tumor site 12 and 24 h following intravenous administration [8]. In recent times, nanoparticles have been increasingly utilized as an alternative approach to conventional vaccines. With significant progress in chemical and biomedical engineering, nanoparticle-based vaccines have been designed to enhance antigen presentation and enhance immunogenicity. The recent approval and widespread adoption of nanocarrier-based COVID-19 vaccines has demonstrated the immense potential of nanomedicine in impactful applications [9,10].

Despite the rapid growth of nanotechnology, only a small number of nanoparticle-based formulations have so far advanced into clinical practice [11]. One major limitation to translation is the lack of comprehensive analysis of nanoparticle cytotoxicity, given the complexity in the nanocarrier design and the potential contributions of a range of physicochemical characteristics, including composition, size, shape, surface charge, and roughness [12]. It is frequently reported that commonly used nanocarrier platforms encounter unresolved cytotoxicity issues during their development. For example, positively charged nanoparticle platforms, such as gold, quantum dots, and dendrimers, penetrate the negatively charged cell membrane but leave behind membrane pores, resulting in cell death [13]. Aluminum oxide nanocarriers induce cytotoxicity by promoting oxidative stress, damaging mitochondrial function, and altering protein expression across the blood–brain barrier [14]. Copper oxide nanoparticles were also found to decrease cell viability via cellular membrane damage and increased lipid peroxidation in human lung epithelial cells [15]. Thus, a comprehensive assessment of nanotoxicology is imperative prior to clinical translation.

Recently, we proposed a novel multifunctional carbon-based nanocarrier, plasma polymerized nanoparticles (PPNs), which are synthesized from reactive dusty plasmas [16]. Fundamental research in dusty plasmas over the last few decades has been largely driven by industry demands, mostly with the aim of suppressing sample contamination caused by the formation of plasma dust nanoparticles. The unintended growth of dust nanoparticles severely compromises the yield and overall process efficiency, particularly in reactive plasma etching, lithography, and semiconductor manufacturing [17]. While dusty plasmas are considered an unwanted impurity in manufacturing industries, the potential of PPNs in nanomedicine was entirely overlooked. The ability to produce nanoparticles in a wet-chemistry-free plasma environment represents a cost-effective synthesis approach, with the ability to easily select specific physical and chemical nanocarrier properties. PPNs are amorphous, carbon-based plasma polymers containing long-lived surface functional moieties that allow for a robust attachment of a wide range of bioactive molecules via surface contact in a simple one-step conjugation protocol. The synthesis and collection of PPNs in plasma reactors are highly efficient and cost-effective [18], resulting in high nanoparticle yields (80 mg/h) in a single-step manufacturing process. PPNs are synthesized under similar plasma polymerization conditions as plasma polymer coatings, which is a process that produces biocompatible interfaces that are suitable for cell- and blood-contact applications [19,20,21]. The therapeutic potential of PPNs has recently been demonstrated, both in vitro and in vivo [22]. PPNs effectively delivered functional siRNA against vascular endothelial growth factor (siVEGF) in hard-to-transfect human coronary endothelial cells, resulting in significantly higher transfection rates than commercial lipofectamine. In a model of breast cancer in mice, multifunctional PPNs modulated the tumor microenvironment and impaired tumor growth upon the delivery of a dual siVEGF and paclitaxel formulation. While PPNs represent an exciting new nanocarrier platform, their broad cytotoxic profile and biosafety in vivo has not been fully characterized.

In this study, we comprehensively assessed, for the first time, the toxicity and biosafety of PPNs in vitro and in vivo. We showed that PPNs did not inhibit cell proliferation nor induce cytotoxicity across the eight cell types tested, including primary cells and cancerous cell lines. Nanoparticle size-dependent toxicity was negligible over a wide range of tested PPN concentrations when compared to a commercially available lipid-based nanoparticle (lipofectamine). Moreover, the intravenous administration of PPNs was physiologically well tolerated in BALB/C mice without any evidence of systemic toxicity, even at the highest dose of 48,000 mg/kg (mass of PPNs/animal weight). These findings highlight a high degree of safety in both cell and animal models, supporting the ongoing development of PPNs as a novel nanotherapeutic platform. 

## 2. Materials and Methods 

### 2.1. Preparation of the PPNs 

The PPNs were synthesized in reactive dusty plasmas via plasma polymerization from acetylene, nitrogen, and argon using low pressure (150 mTorr), radiofrequency discharges (13.52 MHz), as previously described [16]. Two nanoparticle formulations were synthesized in this study to assess the toxicity and biosafety profiles of the PPNs with an average diameter of ≈130 nm (PPN1) and ≈230 nm (PPN2). The plasma parameters, including the input power, gas flow rates, and discharge pressure, were chosen based on our previous studies [16,18] to produce similar surface chemistry profiles but different nanoparticle sizes. The PPNs were collected from the plasma discharge using 24-well polystyrene plates (CLS3524, Merck, Darmstadt, Germany), as previously described [18]. This method allowed for the controlled collection and post-synthesis handling of high yields of PPNs with well-defined physical and chemical properties and without inducing nanoparticle aggregation. The nanoparticles were then resuspended directly from wells under sterile conditions with RT-PCR-grade water (4387936, Life Technology, Carlsbad, CA, USA).

### 2.2. Physical and Chemical Characterization of the PPNs 

The hydrodynamic size and zeta potential of the PPNs were measured using a dynamic light-scattering Zetasizer Nano ZS system (Malvern Panalytical, Almelo, The Netherlands), as previously described [16]. Briefly, the PPNs were dispersed in RT-PCR-grade water and transferred to disposable folded capillary cells (DTS1070, Malvern Panalytical, Almelo, The Netherlands). The sizes and zeta potentials were obtained from the average of five measurements at room temperature and pH = 6.5. To obtain the relative surface elemental compositions of the PPNs, we carried out X-ray photoelectron spectroscopy (XPS) using a Bruker K-Alpha X-ray spectrometer (Bruker, Billerica, MA, USA). The PPNs were collected directly from the plasma discharge onto 5 × 5 mm^2^ (111) type p (boron) silicon wafers (Proscitech, Kirwan, Australia) placed on center wells of the 24-well polystyrene plates and were stored at room temperature for 15 days before the XPS measurements. The relative atomic fractions (at%) of carbon, nitrogen, and oxygen were determined by calculating the integrated areas of the C1s, N1s, and O1s core–shell peaks and assuming that they summed to 100% (i.e., disregarding elemental hydrogen). Data analysis was performed using Avantage V5.9922 software (Thermo Scientific, Waltham, MA, USA). SEM was performed using a Zeiss Sigma HD FEG (Zeiss, Oberkochen, Germany) scanning electron microscope at an acceleration voltage of 5 kV and a working distance of 10 mm. 

### 2.3. Cell Viability and Proliferation Assays 

The effect of the PPN formulations on cell viability and proliferation was determined using AlamarBlue^TM^ Reagent (DAL1100, Life Technologies, Carlsbad, CA, USA). Adeno-carcinoma human alveolar basal epithelial cells (A549, 86012804, passage 15–20, Merck, Darmstadt, Germany), human coronary artery cells (hCAECs, 300-05a, passage 4–6, Cell Applications, San Diego, CA, USA), human embryonic kidney cells (HEK293, CRL-1573 passage 15–25, ATCC, Manassas, VA, USA), human malignant epithelial cells (HeLa, 93021013 passage 20–30, Merck, Darmstadt, Germany), human liver cancer cells (HEPG2, 85011430 passage 15–25, Merck, Darmstadt, Germany), human fibroblast (hFB, CSC-C4381X passage 4–6, Creative Bioarray, Shirley, NY, USA), human vascular smooth muscle cells (hSMCs, CSC-C4357X passage 4–6, Creative Bioarray, Shirley, NY, USA), induced pluripotent stem cell-derived endothelial cells (IPSC-ECs, i357-20 passage 13–16, Cell Applications, San Diego, CA, USA), and breast cancer cells (MCF7, 86012803 passage 15–25, Merck, Darmstadt, Germany) were cultured on 96-well plates 24 h prior to the study. The cell types chosen in this work provided insight into the interaction of the PPNs with primary cells, which are known to be more susceptible to nanoparticle toxicity, and cancer cell lines, as many nanoparticle platforms are being developed to improve treatment options in cancer applications. hCAECs and IPSC-ECs were cultured in Mesoendo medium (Merck, 212–500, Kenilworth, NJ, USA) and SMC in smooth muscle cell medium (311–500, Merck, Darmstadt, Germany), while HEK 293, HEPG2, A594, MCF7, and hFB cells were cultured in a mixture of DMEM with 10% FBS. The culture growth medium was removed and replaced with a fresh medium containing 100 µL of the PPNs at concentrations ranging from 10^3^ to 10^9^ particles per mL. Cells receiving growth medium only (NTC) were used as a negative control, whereas lipofectamine-treated cells (Lipofectamine RNAiMax, ThermoFisher Scientific, Waltham, MA, USA) were used as a nanocarrier control group (as per the manufacturer’s instructions). The cells were incubated with the PPNs at 37 °C in 5% CO_2_. At pre-determined time points (days 3 and 7), the growth medium was removed and 90 µL of fresh growth media and 10 µL of the AlamarBlue^TM^ reagent were added into each well. After 2 h of incubation with the reagent, the AlamarBlue^TM^ fluorescence was then measured using a CLARIOstar microplate reader. The relative cell viability and the percentage of proliferation were calculated and normalized against the NTC group. 

### 2.4. Cell Membrane Integrity Assay 

The cell membrane integrity was measured using a lactate dehydrogenase LDH leakage assay. LDH, which is a soluble enzyme located in the cytosol, is released into the cell culture medium upon cell membrane damage, where it can be quantified due to the coupling enzymatic reactions of lactate conversion to pyruvate and NAD+ reduction to NADH (88953, Life Technologies, Carlsbad, CA, USA). After 3 days of incubation with the PPNs (10^7^ to 10^9^ particles/mL), 50 µL of culture medium was collected and transferred into a new 96-well plate. NTC was used as a negative control, whereas 0.2% *v*/*v* Triton X-100 (X-100, Merck, Darmstadt, Germany) treated cells were used as a positive control. The lipofectamine-treated cells (as per the manufacturer’s instruction) were used as a nanocarrier control group. All the control samples were collected in the same manner. LDH (100 µL) was then added to all the groups. All samples were then incubated for 30 min at room temperature. The LDH activity (measured at 490 nm) was then measured using a CLARIOstar microplate reader and the percentage of membrane disruption was calculated using the following equation: cytotoxicity (%) = [(S − MC)/(T − MC)] × 100, where S, MC, and T are the absorbance of test samples, tissue culture media controls, and Triton X-100 control, respectively.

### 2.5. Apoptosis Assay 

The apoptotic activity was assessed using Annexin V-FITC/propidium iodide (PI) double-staining (APOAF-20TST, Merck, Darmstadt, Germany). The cells (1 × 10^5^) were seeded into a 12-well plate and treated with the PPN1 (10^10^ particles/mL) and PPN2 (10^9^ particles/mL) formulations. The NTC and Triton-X-100-treated cells were utilized as control groups. Following incubation of 24 h, the cells were harvested and stained, as per the manufacturer’s instructions. All samples were incubated for 15 min at 37 °C with 5 µL Annexin V-FITC and 5 µL PI diluted in 490 µL of binding buffers (50 mM HEPES, 700 mM CaCl_2_, pH 7.4). Apoptotic and necrotic cells were immediately analyzed via flow cytometry (BD FACSVerse). The analysis was then performed on FACSuite^TM^ Version 8.0 software with cell populations selected using a forward scatter (FSC)/sideward scatter (SSC) dot plot, thereby excluding cell debris (Figure S1). The population gate was set for 50,000 events. These gated events were then further analyzed for Annexin V (FITC channel, laser 488 nm) and PI (PE channel, laser 617 nm). The percentage of cells in each quadrant was then recorded. The lower-left (LL) quadrant consisted of the viable population, while necrotic cells were observed in the upper-left (UL) and the upper-right (UR) quadrants. The apoptotic cells were distinguished in the lower-right (LR) quadrant.

### 2.6. In Vivo Biosafety Studies 

BALB/c mice (male, 7 weeks of age, body weight between 20–22 g) were selected for the study. Ethics approval was obtained from the Sydney Local Health District Animal Welfare Committee (AWC), protocol number 2015/041. The experiments were conducted in accordance with the Australian Code of Practice for the Care and Use of Animals for Scientific Purposes. All personnel involved in the animal procedures have completed an approved animal care and ethics course.

#### 2.6.1. Acute Dose

The BALB/c mice were divided into three groups: (1) saline control, (2) PPN1 formulation at the concentration of 60 mg/kg, and (3) PPN1 at the concentration of 6000 mg/kg. PPN1 group (200 µL in saline) was administrated intravenously via a tail-vein injection.

#### 2.6.2. Repeated Doses 

Mice were divided into four groups: (1) saline control and PPN1 formulations at the total concentrations of (2) 60 mg/kg, (3) 6000 mg/kg, and (4) 48,000 mg/kg. The PPN1 groups (200 µL in saline) were intravenously injected into the mice via a tail vein. The total PPN1 dosage was equally divided into four injections at days 0, 3, 6, and 8. The body weight of all mice was monitored for 14 days for both the acute and repeated dose studies. At the chosen time points, blood samples (500 µL) were collected in EDTA tubes using a cardiac puncture technique for toxicity screening, complete blood count (CBC), and biochemical measurements. The blood and biochemical analysis were independently obtained from Vetnostics Laboratory (Sydney, Australia). Whole animal perfusion fixation was performed, and the organs were collected for histology studies. 

### 2.7. Histological Studies 

Organs, including heart, spleen, liver, kidneys, and lung, were collected and fixed in 10% buffer formalin (HT5014, Merck, Darmstadt, Germany) and embedded in paraffin. Paraffin-embedded tissue sections (5 µm thickness) of all organs were prepared and stained with hematoxylin and eosin (H&E). 

### 2.8. Data Analysis 

All statistics are displayed as mean ± standard deviation (SD) with five repeats in all tested samples (*n* = 5), unless specified otherwise. Ordinary one-way analysis of variance (ANOVA) followed by Dunnett’s multiple comparison test was applied for multiple comparisons of three or more group means. *p* < 0.05 was considered statistically significant. ****, ***, **, and * represent *p* < 0.0001, *p* < 0.001, *p* < 0.01, and *p* < 0.05, respectively.

## 3. Results

### 3.1. PPN Synthesis and Characterization

**Figure 1:**
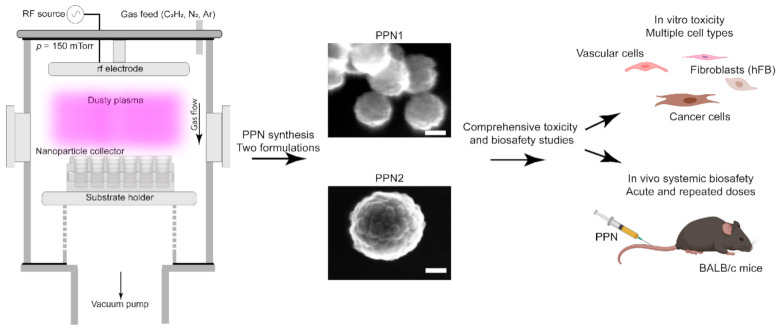
Schematic depicting the overall goal of this work. Two PPN formulations were synthesized in acetylene-containing dusty plasmas for evaluation of their cytotoxicity in various cell types and systemic biosafety in BALB/c mice. Scale bars represent 50 nm.

The size of nanocarriers is a key physical property that governs its toxicity [23]. In this work, we assessed the cytotoxicity and biosafety of two PPN formulations with distinct sizes, which were synthesized in acetylene-based reactive dusty plasmas (Figure 1). Dry synthesis in a plasma medium allows for tailoring the nanoparticles’ physical and chemical properties by adjusting key plasma generation parameters (e.g., discharge pressure and input power) and chemistry (e.g., gas type and flow rates) [16,24]. Here, plasma parameters (Table 1) were adjusted to produce PPNs with two sizes while maintaining their surface charge and chemistry to be as close as possible. For both formulations, the discharge pressure was kept constant at 150 mTorr, and the flow rates of nitrogen and argon were set to 10 sccm and 3 sccm, respectively. The first set yielded a PPN formulation (PPN1) with an average hydrodynamic diameter of 234 ± 32 nm (345 ± 56 nm in PBS + 10% FBS) while using an input power of 50 W and a C_2_H_2_ flow rate of 3 sccm. Smaller PPNs (PPN2), with an average hydrodynamic diameter of 128 ± 23 nm (212 ± 23 nm in PBS + 10% FBS), were synthesized by increasing the input power to 100 W and the C_2_H_2_ flow rate to 6 sccm. The resulting PPN formulations were positively charged (ζ = +36 mV for PPN1 and ζ = +37 mV for PPN2) when dispersed in ultrapure water (pH = 6.5) but negatively charged (ζ = −8 mV for PPN1 and ζ = −22.9 mV for PPN2) when dispersed in a PBS buffer with 10% FBS (pH = 7.2). Both formulations were characterized by an elemental ratio of C/N ≥ 2.1 and a relative O content of 10% 15 days after their synthesis (the latter was due to surface oxidation). Variations in the relative C, N, and O elemental compositions between both formulations were below 3.4% (Table 1).

### 3.2. In Vitro PPN Cytotoxicity

A dose- and size-dependent cytotoxicity assessment of the PPNs was performed across eight different cell types, representing two major classes of cells, namely, primary cells (hFBs and vascular cells (hCAECs, IPSC-ECs, hSMCs)) and cancer cells (HEPG2, MCF7, A549, and HeLa), using three separate assays: (1) cell proliferation, (2) cell membrane integrity, and (3) cell apoptosis and necrosis.

#### 3.2.1. Evaluation of the PPNs’ Cytotoxicity on Primary Cells 

**Figure 2:**
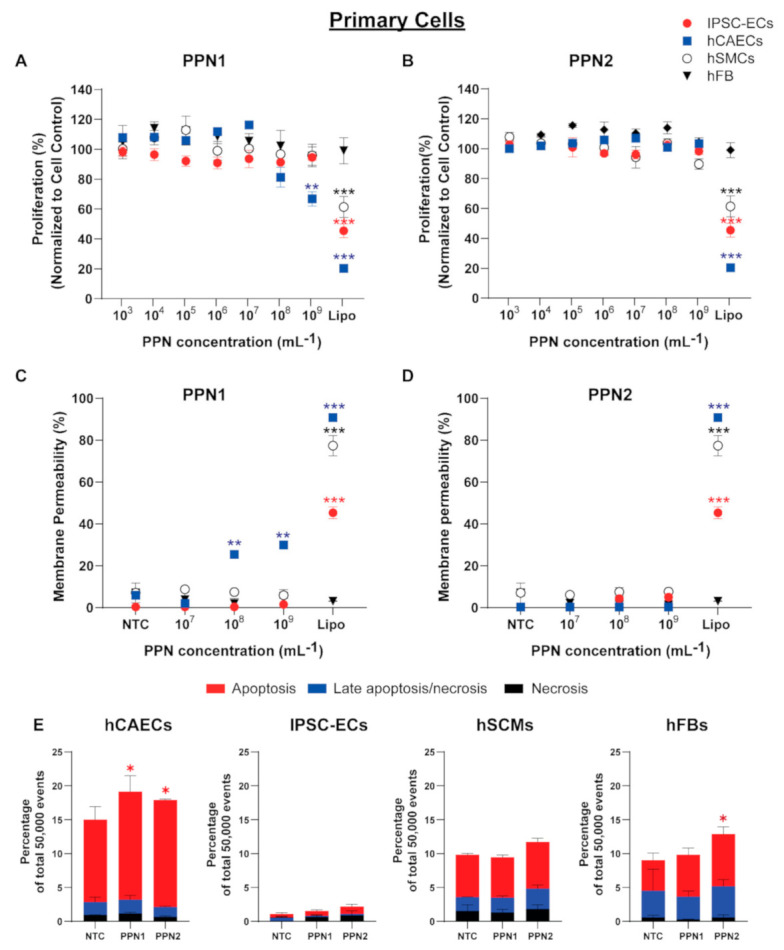
The effect of the size (PPN1 and PPN2) and dose of PPNs (concentration ranging 10^3^–10^9^ PPN/mL on (**A**,**B**), cell proliferation, (**C**,**D**) cell membrane integrity, and (**E**) apoptosis in primary cells (IPSC-ECs, hCAECs, hSMCs, and hFBs). Untreated cells (NTC) and cells treated with lipofectamine were used as control groups. The data represent the mean ± standard deviation (*n* = 4). Statistical significance was determined using Tukey’s multiple comparison test (***, ** and * represent *p* < 0.001, *p* < 0.01 and *p* < 0.05, respectively).

We first assessed the effects of both PPN formulations on the proliferation of vascular cells (hCAECs, IPSC-ECs, hSMCs) and hFBs. All cell types were exposed to PPNs at concentrations ranging from 10^3^ to 10^9^ PPN/mL for 3 and 7 days. No significant changes in the proliferation of the IPSC-ECs, hSMCs, and hFBs were observed following incubation with the PPN1 formulation at all concentrations and at both time points tested here (Figure 2A). However, a reduction in the hCAECs proliferation was observed compared to the control cells (NTC) for cells receiving PPN doses of 10^8^ PPN/mL (81.25 ± 14.25%) and 10^9^ PPN/mL (66.79 ± 10.74%). In contrast, the PPN2 formulation had no effect on cell proliferation in any of the cell types (Figure 2B). A commercially available lipid-based nanocarrier lipofectamine was used as a positive nanocarrier control group. At the manufacturer’s suggested concentration (Lipofectamine RNAiMax 1.5 µL/well, 96-well plate), lipofectamine significantly reduced cell proliferation of hCAECs (20.35 ± 0.50%), IPSC-ECs (45.40 ± 6.49%), and hSMCs (61.39 ± 7.09%), while hFBs grew comparably to control (NTC) at 99.02%. 

Next, we assessed the cellular membrane integrity using a lactate dehydrogenase leakage assay (LDH). Consistent with results from the cell proliferation assay, PPN1 treatment did not induce an LDH release in IPSC-ECs, hSMCs, or hFBs (Figure 2C). Meanwhile, a significant increase in release of LDH was observed in hCAECs treated with PPN1 at 10^8^ PPN/mL (25.37 ± 1.94%) and 10^9^ PPN/mL (29.96 ± 2.78%) when compared to cell control (5.9 ± 0.82%). All vascular cells and hFBs that were exposed to the PPN2 formulation did not exhibit significant changes in LDH activity compared to the untreated cells (Figure 2D). In contrast, application of the lipofectamine led to a significant increase in LDH activity in hCAECs (90.82 ± 2.19%), IPSC-ECs (45.28 ± 4.03%), and hSMCs (77.33 ± 6.86%), but not in hFBs (3.03 ± 0.10%), 72 h following incubation.

We next examined the degree of cell apoptosis and necrosis (Figure 2E). Vascular cells were incubated with both PPN formulations at the highest concentration of 10^9^ PPN/mL for 24 h and stained with Annexin V-FITC (an early apoptotic marker) and propidium iodide (a necrotic marker). Quantitative flow cytometry analysis revealed only a slight increase in apoptotic activity in hCAECs (15.99 ± 1.89% for PPN1 and 15.77 ± 0.12% for PPN2) and hFBs (7.70 ± 0.89% for PPN2) when compared to the NTC (12.19 ± 1.56% for hCAECs, 4.53 ± 0.83% for hFBs). However, no significant differences in the fractions of apoptotic cells (compared to NTC) were observed in IPSC-ECs (9.39 ± 0.64% for PPN1 and 10.78 ± 0.46% for PPN2), hSMCs (5.91 ± 0.28% for PPN1 and 6.91 ± 0.42% for PPN2), and hFBs (6.2 ± 0.80% for PPN1) following PPN exposure.

#### 3.2.2. Evaluation of the PPNs’ Cytotoxicity on Cancer Cells

**Figure 3:**
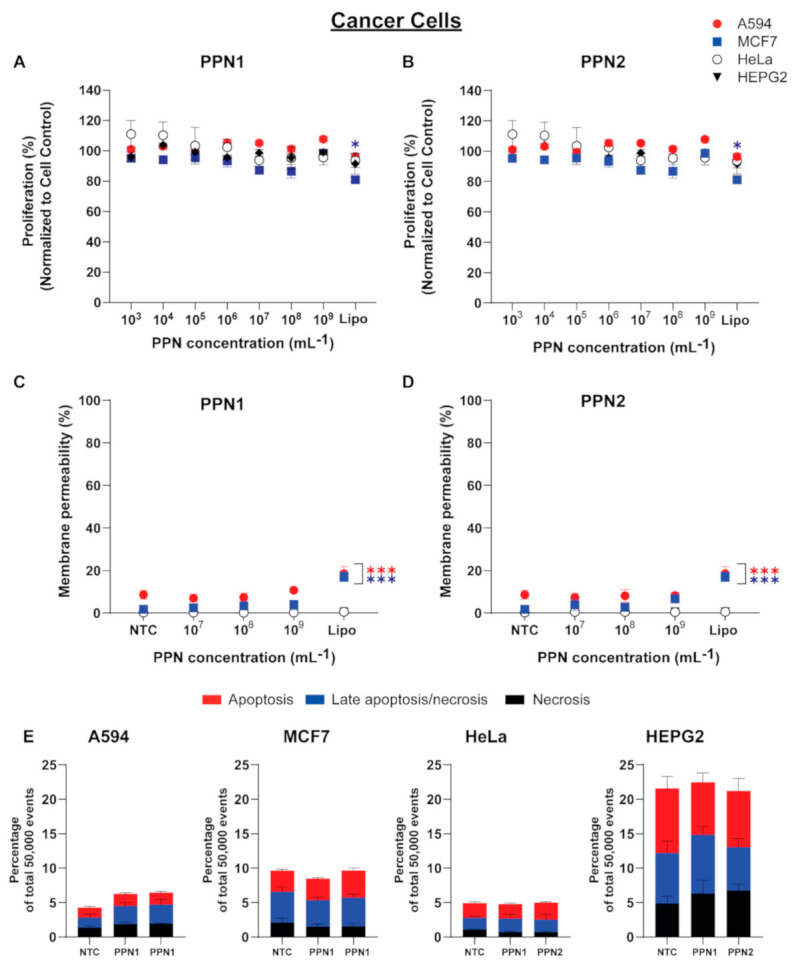
The effects of the size (PPN1 and PPN2) and dose of the PPNs (concentration ranging from 10^3^ to 10^9^ PPN/mL on (**A**,**B**) cell proliferation, (**C**,**D**) cell membrane integrity, and (**E**) apoptosis in cancer cells (A594, MCF7, HeLa, and HEPG2). Untreated cells and cells treated with lipofectamine were used as control groups. The data represent the mean ± standard deviation (*n* = 4). Statistical significance was determined using Tukey’s multiple comparison test (*** and * represent *p* < 0.001 and *p* < 0.05).

The effects of PPNs on cell proliferation, membrane integrity, and apoptosis in A594, HeLa, HEPG2, and MCF7 were also investigated. In general, cell proliferation was not significantly affected by either PPN formulation at concentrations ranging from 10^3^ to 10^9^ PPN/mL (Figure 3A,B). In comparison, lipofectamine significantly inhibited MCF7 growth, reducing its proliferation to 81.09 ± 2.19% compared to NTC, while the remaining cancer cells were not affected by lipofectamine. A similar trend was observed in the cell membrane integrity LDH assay for all tested cancer cells, where the LDH activity remained comparable to NTC for both PPN formulations (Figure 3C,D). The LDH activity was significantly increased in lipofectamine-treated groups, particularly for A594 (17.07 ± 3.17%) and MCF7 (18.05 ± 4.81%). Furthermore, the fraction of apoptotic cells in all cell types treated with both PPN formulations was comparable to NTC (Figure 3E).

### 3.3. Toxicity and Biosafety of PPNs In Vivo 

Whilst in vitro cell-based systems allow for a comprehensive study of cell-specific effects, in vivo animal models offer an insight into the effects of nanoparticle exposure to the whole-body system. In this study, we chose a commonly used BALB/c mice model to investigate systemic PPN toxicity. Acute- and repeated-dose toxicity studies were performed to assess the physiological safety.

#### 3.3.1. Acute-Dose Toxicity 

**Figure 4:**
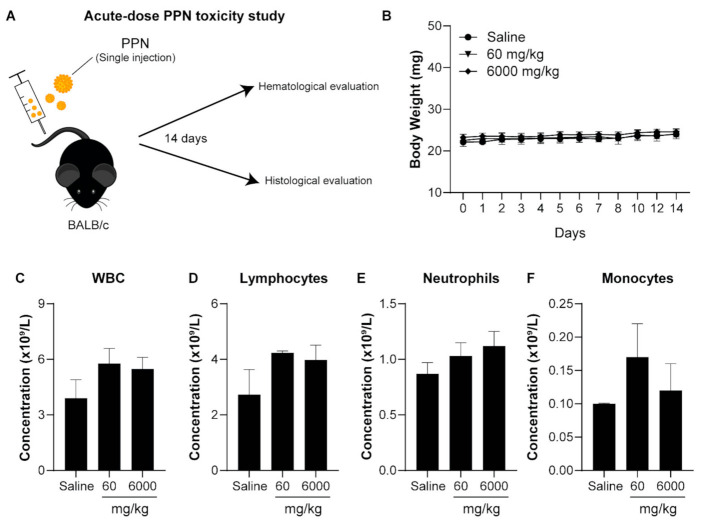
(**A**) Experimental schematic depicting the acute toxicity dosing of the PPNs in BALB/c mice via a single intravenous tail vein injection, followed by blood and organ analysis after 14 days. (**B**) Body weight of BALB/c mice receiving either saline or PPN doses of 60 and 6000 mg/kg over 14 days post-injection. Hematology analysis consisting of (**C**) white blood cell, (**D**) lymphocyte, (**E**) neutrophil, and (**F**) monocyte counts at 14 days post-injection. The data represent the mean ± SEM of five mice.

The mice were given single PPN1 doses intravenously at concentrations of 60 mg/kg and 6000 mg/kg to assess the systemic toxicity of PPN1 in an acute-dose regime (Figure 4A). Here, we chose to study the biosafety of PPN1 since this was the only formulation that induced mild toxicity in hCAECs in our in vitro studies. A group receiving saline injections was utilized as the control. Mice were monitored for 14 days for changes in their weight and behavior prior to terminal blood sampling and histology. The survival rate of the mice was sustained at 100% for the duration of the study. No reduction in body weight was observed in mice following either 60 mg/kg or 6000 mg/kg intravenous injection of PPNs (Figure 4B). No signs of swelling, redness, pain, or heat at the site of the injection were detected in any mice at either concentration. Furthermore, no discomfort or stress behaviors were detected following exposure to both PPN dosages for the entire duration of the study. 

**Figure 5:**
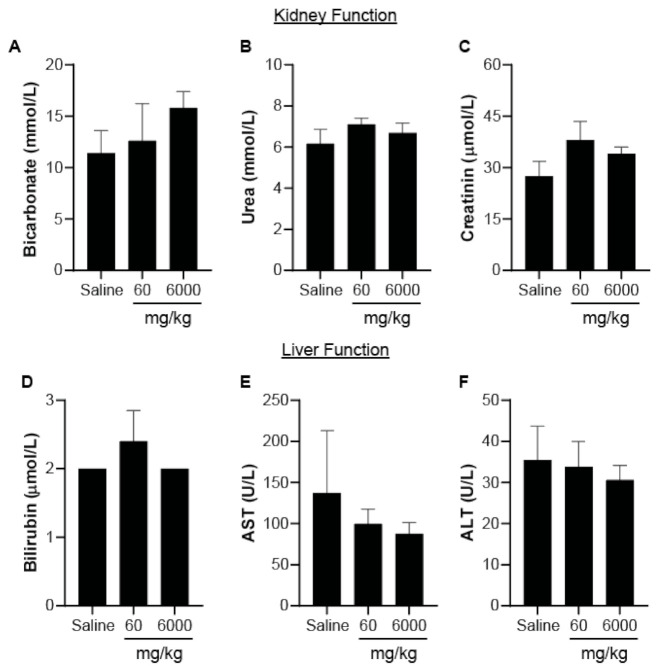
Hematology analysis of the effects of PPNs (acute-dose toxicity) on kidney and liver function. (**A**) Bicarbonate, (**B**) urea, (**C**) creatinine, (**D**) bilirubin, (**E**) AST, and (**F**) ALT levels in mice plasma 14 days after the PPN (60 mg/kg and 6000 mg/kg) or saline injection. The data represent the mean ± SEM of five mice.

Upon sacrifice, blood samples were collected for independent hematological and blood biochemical analysis in accordance with previous studies [25]. For the hematological toxicity, we analyzed the concentration of hemoglobin (Hb), red cell count (RCC), hematocrit (Hct), mean corpuscular volume (MCV), mean corpuscular hemoglobin (MCH), mean corpuscular hemoglobin concentration (MCHC), and platelet counts. We observed no significant changes in any of these markers following PPN injections relative to mice that received saline only (Table 2). The number of white blood cells (WBC), including neutrophils, lymphocytes, monocytes, eosinophils, and basophils, was also investigated to assess any inflammatory response induced by PPNs. Again, no significant changes in the levels of the immune cells compared to the saline control were observed (Figure 4C–F). Furthermore, analysis of the blood biochemistry profile revealed that kidney function (indicated by the levels of bicarbonate, urea, and creatinine) and liver function (indicated by the levels of bilirubin, AST, and ALT), as well as pancreas function and cholesterol levels in mice treated with the PPNs, were equivalent to the saline control mice (Figure 5A–F) and appeared to be within the normal range for BALB/c mice (Table 3). Together, these results indicate that an acute, single intravenous injection of PPNs, even at high concentrations in mice, did not induce any hematological toxicity.

**Figure 6:**
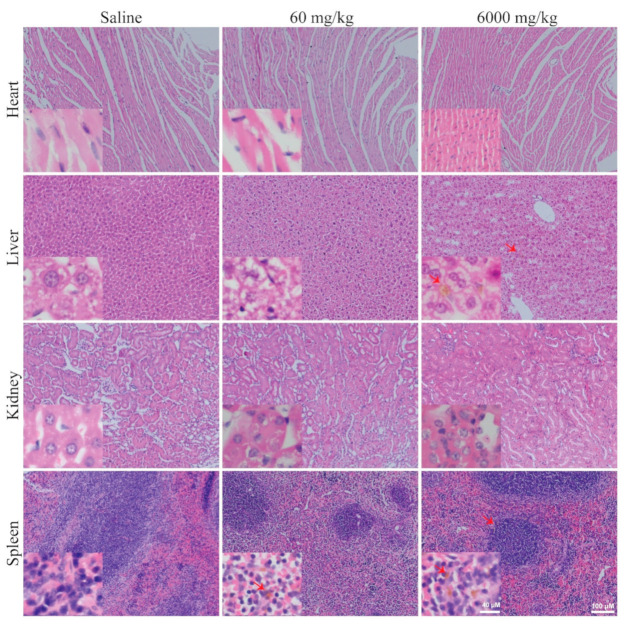
Histopathology of mice organs receiving a single PPN dosage at the concentration of 60 or 6000 mg PPNs/kg after 14 days post single IV administration. Sparse, small depositions (≤20 µm) of PPNs were observed in the liver and spleen (red arrow). No major abnormalities in the organ structure were detected for both PPN concentrations. Scale bars represent 40 µm (insets) and 100 µm.

Pathohistological evaluation of organs, including heart, liver, lung, kidney, and spleen, from mice injected with PPNs were also performed to determine the organ structural abnormalities (Figure 6 and Figure S2). H&E staining showed no major morphological change in any of the organs (Figure 6), whereas trace accumulation of PPNs was observed mainly in the liver and spleen after 14 days post-injection. Together, both histological and blood toxicity analysis suggest that PPN are well tolerated.

#### 3.3.2. Repeated-Dose Toxicity

**Figure 7:**
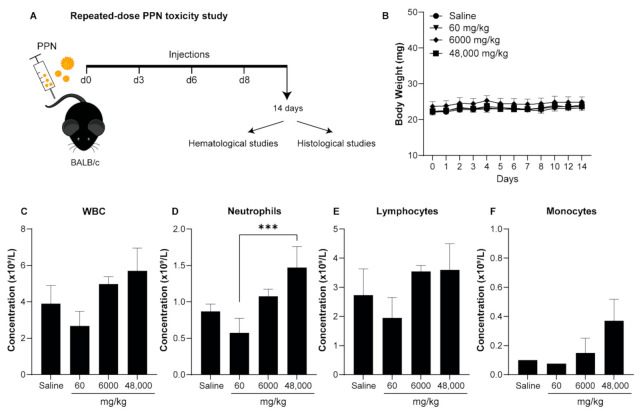
(**A**) Schematic depicting the experimental procedure to evaluate the toxicity of PPNs in BALB/c mice upon repeated doses. (**B**) No reduction in body weight was observed after IV administration of repeated doses of PPNs for the entire duration of the study (14 days). A slight elevation of (**C**) WBC, (**D**) neutrophils, (**E**) lymphocytes, and (**F**) monocytes was observed at the higher doses (6000 mg PPN/kg and 48,000 mg PPN/kg) when compared to the saline control group. The data represent the mean ± SEM of five mice and the statistical significance was determined using Tukey’s multiple comparison test (*** *p* < 0.001).

To investigate the toxicity of the PPN1 formulation under a repeated-dose regime, we performed four tail vein injections at different concentrations over 14 days (Figure 7A). The accumulated final concentrations for each group were 60 mg/kg, 6000 mg/kg, and 48,000 mg/kg. The mice survival rate receiving the repeated injections of PPN was 100% for all dosages for the entire duration of the study. Repeated injections of PPNs did not induce any significant changes in the mice’s body weights compared to the saline control (Figure 7B). No discomfort or stress behaviors were observed following the injection of PPNs for all concentrations tested.

**Figure 8:**
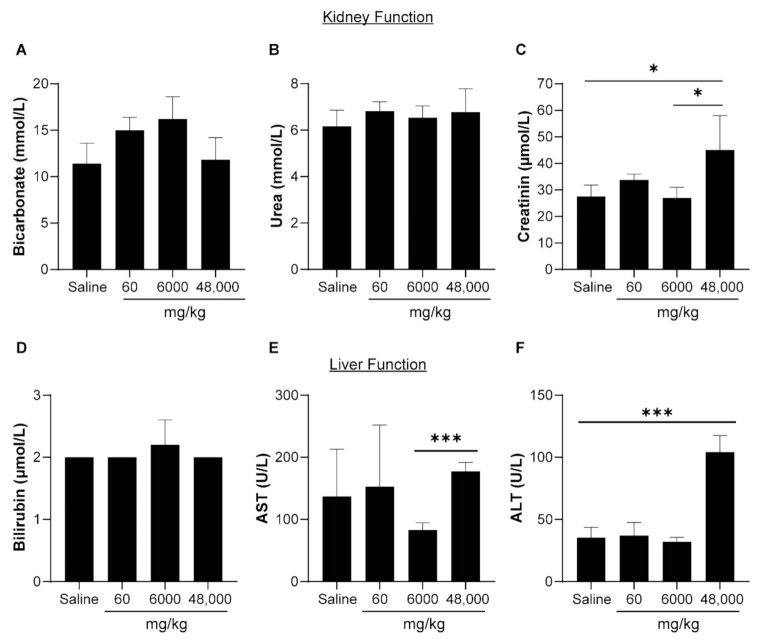
Effects of PPNs (repeated doses) on kidney and liver function. (**A**) Bicarbonate, (**B**) urea, (**C**) creatinine, (**D**) bilirubin, (**E**) AST, and (**F**) ALT levels in the mice plasma 14 days after the PPN (60 mg/kg, 6000 mg/kg and 48,000 mg/kg) or saline injections. The data represent the mean ± SEM of five mice and the statistical significance was determined using Tukey’s multiple comparison test (*** and * represent *p* < 0.001 and *p* <0.01, respectively).

**Figure 9:**
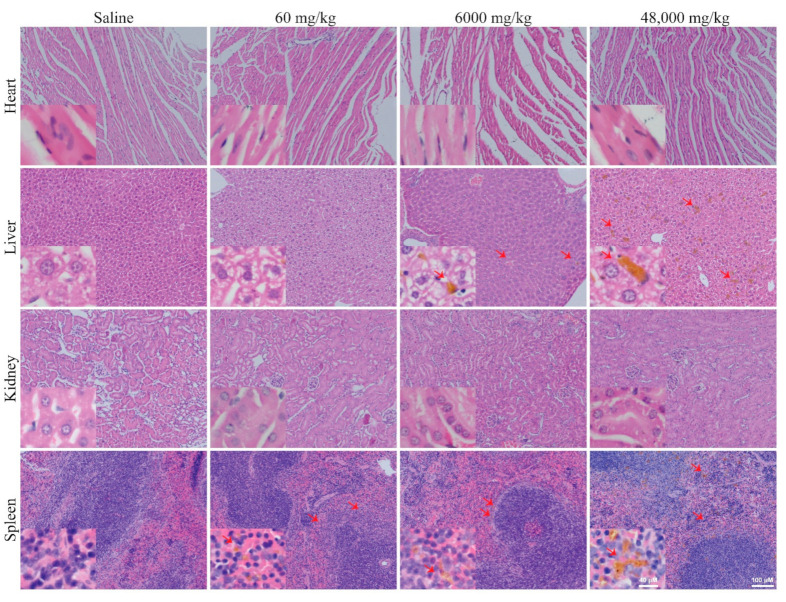
Histopathology of mice organs receiving accumulated concentrations of 60, 6000, and 48,000 mg/kg after 14 days following the first IV administration. Sparse PPN deposits (≤100 µm) were observed mainly in the liver and spleen at the higher dosages, as indicated by the red arrows. No major abnormalities in organ structure were detected for all the PPN concentrations tested. Scale bars represent 40 µm (insets) and 100 µm.

The hematological profile showed that the levels of Hb, RCC, hCT, MCV, MCH, and MCHC of the RBCs in the mice that received four repeated doses of PPNs remained within the normal range or comparable to the saline control mice, suggesting that the repeated administration of PPNs did not induce significant hematological toxicity (Table 4). There was a moderate increase in the total WBC, neutrophil, and monocyte counts upon the repeated injection of higher doses, though this did not reach statistical significance compared to the saline control (Figure 7C–F). The levels of bicarbonate and urea were not significantly different between the PPN treatment groups and saline controls (Figure 8A,B). However, the levels of creatinine were significantly higher in mice receiving four injections of PPN for a total dose of 48,000 mg/kg (Figure 8C). However, we note that this dose is extremely high and several orders of magnitude above a potential therapeutic dose. The liver function indicated by the levels of bilirubin and AST were comparable between groups (Figure 8D,E). However, there was a threefold increase in the ALT levels in mice receiving the highest dose (48,000 mg/kg) compared to the saline control group at day 14 (Figure 8F). All other parameters analyzed in the blood biochemistry profile remained normal (Table 5). H&E staining to the heart, liver, kidney, spleen, and lungs revealed no morphological changes in any of the PPN groups compared to the saline control (Figure 9 and Figure S2). 

## 4. Discussion

Nanoparticle toxicity represents a major barrier to biomedical utility and ultimately clinical translation of nanocarriers. Therefore, a comprehensive evaluation of the toxicity and biosafety profiles of nanoparticle platforms is an essential step. Following on from our previous work, which proposed nanoparticles synthesized in carbonaceous dusty plasmas (PPN) as a versatile nanocarrier, we recently demonstrated the therapeutical potential of PPN as an effective nanocarrier for the delivery of chemotherapeutic drugs and siRNA in an established cancer model [22]. While the formation mechanisms and physicochemical properties of carbonaceous dusty particles are well reported, their interaction with biological systems, particularly their toxicity, has not yet been fully characterized. This study provides the first comprehensive evaluation of the toxicity of PPNs using both in vitro cell-based models and in vivo mouse models. 

Given that nanoparticle size and dose are critical contributing factors governing cytotoxicity, we first investigated the interaction of two different sizes of PPNs, namely ≈130 nm (PPN1) and ≈230 nm (PPN2), in vitro at concentrations ranging from 10^3^ to 10^9^ PPN/mL with various cell types seeded in a surface area of 0.32 cm^2^. The broad range of nanoparticle concentrations and cell types selected in this study are comparable to other in vitro nanotoxicity studies on commercially available nanoparticles [12,25,26]. Overall, we demonstrated that PPNs did not inhibit proliferation of hFBs, vascular cells (IPSC-ECs, hSMCs), or cancer cell lines (A594, MCF7, HeLa, and HEPG2), independent of the nanoparticle size, even at higher concentrations (≤10^9^ PPNs/mL). Inhibition in hCAECs’ proliferation was observed at the highest PPN1 concentration (≥10^8^/mL), while PPN2 was well tolerated at all concentrations. It is expected that the cytotoxic effects of nanoparticles are highly dependent on the type of cell involved. Cancer cells have a higher rate of proliferation and metabolic activity than primary cells and, therefore, can tolerate higher nanoparticle toxicity [27]. Furthermore, the results obtained from the LDH assay and Annexin-V/PI flow cytometry analysis were well corroborated with the cell proliferation assay. Incubation with PPN1 at the highest dose (10^9^ PPN/mL) led to an increase in LDH activity (>20%) and apoptosis rate (>5%) in hCAECs. These results indicate that membrane disruption, a key feature of cells undergoing apoptosis, is a significant cytotoxic mechanism in hCAECs when exposed to smaller PPNs at higher concentrations. Accordingly, recent studies have shown that the effects of zinc oxide nanoparticles (ZnONPs) [28] on hCAECs’ viability are directly proportional to the nanoparticle concentration but inversely proportional to the size [29]. Smaller nanoparticles have a greater surface area per unit volume, thereby increasing their interactions with cellular components that lead to cellular damage [30]. Additionally, smaller-sized nanoparticles diffuse faster than the larger nanoparticles of the same formulation and, therefore, undergo greater cellular uptake. Chithrani et al. [30] reported that 50 nm AuNPs entered the cells more effectively than 100 nm AuNPs. Another study also showed that the uptake rate of silica NPs decreases with increased nanoparticle size [31]. Therefore, small-sized nanoparticles generate a potential trade-off between “cytotoxicity induction” and “effective cellular uptake” that needs to be considered. 

In this study, hCAECs were the most sensitive cell type upon exposure to the smaller PPN1 formulation, which was somewhat expected given that primary human cells are known to be susceptible to cell-penetrating agents and nanoparticles. Interestingly, IPSC-ECs were found to be more resistant to PPN exposure than hCAECs in our study. IPSC-ECs are derived from the reprogramming of somatic fibroblasts into pluripotent stem cells, which are subsequently differentiated into more robust endothelial cells, while hCAECs are somatic cells that are isolated directly from human coronary arteries. Therefore, it is not unexpected that hCAECs and IPSC-ECs responded differently to the same nanoparticle formulation. Nevertheless, we note that hCAECs still tolerated the PPN1 formulation at a high concentration (10^8^/mL) and PPN2 throughout the full concentration range. Given that a PPN is a novel type of nanocarrier, we used lipofectamine to better assess nanotoxicity and compare the PPNs against a commercially available platform. Overall, the levels of PPN toxicity were significantly lower compared to the lipofectamine, importantly in primary cells (hCAECs, IPSC-ECs, and hSMCs), which are known to be susceptible to nanotoxicity. 

Since only the PPN1 formulation exhibited cytotoxicity in hCAECs at higher concentrations, we further investigated the biosafety of this formulation in BALB/c mice. In this study, PPN1 was administrated via intravenous tail vein injection in a single, acute dose regime (60 mg/kg and 6000 mg/kg) and a repeated dose regime (four injections totaling 60 mg/kg, 6000 mg/kg, and 48,000 mg/kg). We note that the dosage range adopted here covers extremely high nanoparticle concentrations, which are significantly higher than the doses that are used to establish the LD_50_ dosages of nanoparticle platforms, including gold nanoparticles (LD_50_ 169.39 ± 1.31 mg/kg) [32] and copper nanoparticles (LD_50_ = 413 mg/kg) [32]. Strikingly, all mice survived the exposure to PPN1, even at the extremely high concentration of 48,000 mg/kg, and it was not possible to establish an LD_50_ value. Furthermore, our results demonstrate that the PPNs were well-tolerated by BALB/c mice, which did not show any signs of weight loss or stress behaviors for the duration of the 14-day study. Similar to other nanomaterials, PPNs accumulated predominantly in the liver, spleen, and kidney, and were absent in cardiac tissues. 

To investigate the systemic effects of PPN1 following IV injection, we conducted an independent and comprehensive analysis of the hematological and biochemical parameters. No significant changes were observed in the hematological profile (Hb, RCC, hCT, MCV, MCH, MCHC, platelets, and WBCs) of the mice treated with PPN1 in either the acute- or repeated-dose toxicity studies. The blood biochemical analysis revealed that the levels of bicarbonate, urea, bilirubin, and AST, which are indicators for liver and kidney function, remained mostly unchanged in all the PPN treatment groups when compared to the parameters of mice that only received saline. Only mice that received repeated administration of the highest dosage (48,000 mg/kg) exhibited elevations of creatinine and ALT in the blood results, suggesting potential kidney and hepatocyte dysfunction. However, we note that the administration of such an extreme nanoparticle dosage would typically result in acute animal death with other platforms and is many orders of magnitude greater than prospective therapeutic doses.

## 5. Conclusions

Plasma-polymerized nanoparticles (PPNs) have recently been proposed as an effective nanocarrier for drug delivery. For the first time, we comprehensively evaluated the toxicity of PPNs using established in vitro cell-based and in vivo mice models. Our findings revealed that the PPNs elicited negligible cytotoxicity and were well-tolerated when administrated via intravenous injection. The low toxicity profile of the PPNs highlighted in this study, compounded with their ease of functionality with molecular cargo and low manufacturing cost, supports the ongoing development of PPNs as a promising therapeutic delivery platform for applications in nanomedicine, including drug delivery, gene therapy, and the treatment of impactful diseases, such as cancer and cardiovascular disease.

## Figures and Tables

**Table 1:** Plasma parameters (radiofrequency input power, gas flow rates, and pressure) that were adopted to synthesize the two PPN formulations studied in this work. The main physical properties (size and zeta potential measured in ultrapure water) and the relative elemental carbon, nitrogen, and oxygen compositions are also shown.

Plasma Parameters						Nanoparticle Properties				
		Gas Flow Rate (sccm)						Elemental Composition (at%)		
Formulation	Power (W)	C_2_H_2_	N_2_	Ar	Pressure (mTorr)	Size (nm)	ζ (mV)	C	N	O
PPN1	50	3	10	3	150	234 ± 32	36 ± 1	61	29	10
PPN2	100	6	10	3	150	128 ± 23	37 ± 1	62	28	10

**Table 2:** Hematological parameters of BALB/c mice 14 days following a single IV administration of PPNs (acute-dose toxicity). The data are expressed as mean ± standard deviation (*n* = 5).

Hematological Profile	PPN Dosage		
	Saline	60 mg/kg	6000 mg/kg
	*n* = 5	*n* = 5	*n* = 5
Hb (g/L)	144.3 ± 6.8	142 ± 6	142.4 ± 2.9
RCC (×10^12^/L)	9.5 ± 0.5	9.57 ± 0.40	9.74 ± 0.17
Hct (%)	45 ± 0	45 ± 2	45 ± 0.1
MCV (fL)	47 ± 1	47.67 ± 0.47	46.8 ± 0.9
MCH (pg)	15 ± 0	15 ± 0.0	15 ± 0
MCHC (g/L)	317.2 ± 6.2	314.33 ± 5.44	313.8 ± 5.0
Platelet (×10^9^/L)	740.7 ± 256.3	889.33 ± 110.76	797.8 ± 202.8

**Table 3:** Blood biochemical serum parameters of BALB/c mice 14 days following a single IV administration of PPN (acute-dose toxicity) on selected. The data are expressed as mean ± standard deviation (*n* = 5).

Blood Biochemistry Profile	PPN Dosage		
	Saline	60 mg/kg	6000 mg/kg
	*n* = 5	*n* = 5	*n* = 5
**Liver/Kidney Function**			
Protein (g/L)	52 ± 5	49.4 ± 2.9	47.6 ± 1.0
Albumin (g/L)	34 ± 1	33.2 ± 1.9	32.6 ± 0.8
Globulin (g/L)	18 ± 4	16.2 ± 1.3	15 ± 1
A/G ratio	1.9 ± 0.3	2.06 ± 0.12	2.18 ± 0.15
Phosphate (mmol/L)	5.0 ± 1.2	4.59 ± 0.20	4.95 ± 0.44
**Pancreas Function**			
Glucose (mmol/L)	6.7 ± 1.9	10.6 ± 3.2	8.94 ± 2.64
**Overall Body Growth, Development, and Health**			
CK (U/L)	4148 ± 1660	3932 ± 99	3812 ± 641
Cholesterol (mmol/L)	3.3 ± 0.3	2.94 ± 0.19	2.84 ± 0.10
Triglyceride (mmol/L)	1.9 ± 0.5	1.58 ± 0.32	1.5 ± 0.1

**Table 4:** Hematological parameters in BALB/c mice following 14 days of repeated IV administration of the PPNs. The data are expressed as mean ± standard deviation (*n* = 5).

Hematological Profile	PPN Accumulated Dosage (Four Injections)			
	Saline	60 mg/kg	6000 mg/kg	48,000 mg/kg
	*n* = 5	*n* = 5	*n* = 5	Average
Hb (g/L)	144.1 ± 6.8	145 ± 7	148.3 ± 2.8	150.8 ± 4.2
RCC (×10^12^/L)	9.5 ± 0.5	9.8 ± 0.5	9.9 ± 0.2	10.4 ± 1.6
Hct (%)	45 ± 0	48 ± 0	50 ± 0	47 ± 1
MCV (fL)	47 ± 1	48.8 ± 0.4	47.8 ± 1.9	46 ± 1
MCH (pg)	15 ± 0	15 ± 0	15 ± 0	15 ± 0
MCHC (g/L)	317.3 ± 6.2	306 ± 2	312.5 ± 11.5	325 ± 9
Platelet (×10^9^/L)	740.7 ± 256.3	768 ± 264	694 ± 162	767 ± 143

**Table 5:** Blood biochemical serum parameters in BALB/c mice following 14 days of repeated IV administration of the PPNs (repeated doses). The data are expressed as mean ± standard deviation (*n* = 5).

Blood Biochemistry Profile	PPN Accumulate Dosage (Four Injections)			
	Saline	60 mg/kg	6000 mg/kg	48,000 mg/kg
	*n* = 5	*n* = 5	*n* = 5	Average
**Liver/Kidney function**				
Albumin (g/L)	34 ± 1	32.2 ± 1.2	32.6 ± 1.2	34 ± 1
Globulin (g/L)	18 ± 4	17.2 ± 4.4	15 ± 1.1	17.2 ± 0.4
A/G ratio	1.9 ± 0.3	1.96 ± 0.4	2.2 ± 0.2	1.98 ± 0.09
Phosphate (mmol/L)	5.0 ± 1.2	6.6 ± 0.6	4.4 ± 0.4	4.87 ± 0.61
**Pancreas function**				
Glucose (mmol/L)	6.7 ± 1.9	15.3 ± 4.8	9.4 ± 3.0	5.9 ± 4.1
**Skeletal muscle injury**				
CK (U/L)	4149 ± 1660	838 ± 712	3565 ± 825	4384 ± 1684
**Overall body growth, development and health**				
Protein (g/L)	52 ± 5	49.4 ± 4.1	47.6 ± 1	51.2 ± 1.5
Cholesterol (mmol/L)	3.3 ± 0.3	2.8 ± 0.2	2.82 ± 0.1	2.8 ± 0.2
Triglyceride (mmol/L)	1.9 ± 0.5	1.5 ± 0.4	1.9 ± 0.5	1.5 ± 0.3

## Supplementary Materials

The following are available online at https://www.mdpi.com/article/10.3390/nano11051176/s1, Figure S1: Flowcytometry data analysis used to determine cell apoptosis and necrosis, Figure S2: Histopathology of mice lungs receiving acute and accumulated PPN dosages.
