# Supplementary material for: Comprehensive Evaluation of the Toxicity and Biosafety of Plasma Polymerized Nanoparticles
Source: Nanomaterials (Basel). 2021 Apr 29;11(5):1176. doi: 10.3390/nano11051176 (PMC8145910; doi:10.3390/nano11051176)
Supplement: Supplementary file 1 [file nanomaterials-11-01176-s001.zip › nanomaterials-1165518-supplementary.pdf]

## Supplementary Material

# Comprehensive Evaluation of the Toxicity and Biosafety of Plasma Polymerized Nanoparticles

**Praveesuda L. Michael [1,2], Yuen Ting Lam [1,2], Juichien Hung [1,2], Richard P. Tan [1,2], Miguel Santos [1,2,*] and Steven G. Wise [1,2,3,*]**

[1] School of Medical Sciences, Faculty of Health and Medicine, University of Sydney, NSW 2006, Australia; praveesuda.michael@sydney.edu.au (P.L.M.); yuen.lam@sydney.edu.au (Y.T.L.); chien.hung@sydney.edu.au (J.H.); richard.tan@sydney.edu.au (R.P.T.)

[2] Charles Perkins Centre, University of Sydney, NSW 2006, Australia

[3] The University of Sydney Nano Institute (Sydney Nano), University of Sydney, NSW 2006, Australia

* Correspondence: miguel.correiadossantos@sydney.edu.au (M.S.); steven.wise@sydney.edu.au (S.G.W.); Tel.: +61-2-8627-9458 (M.S.); +61-2-8627-9458 (S.G.W.)

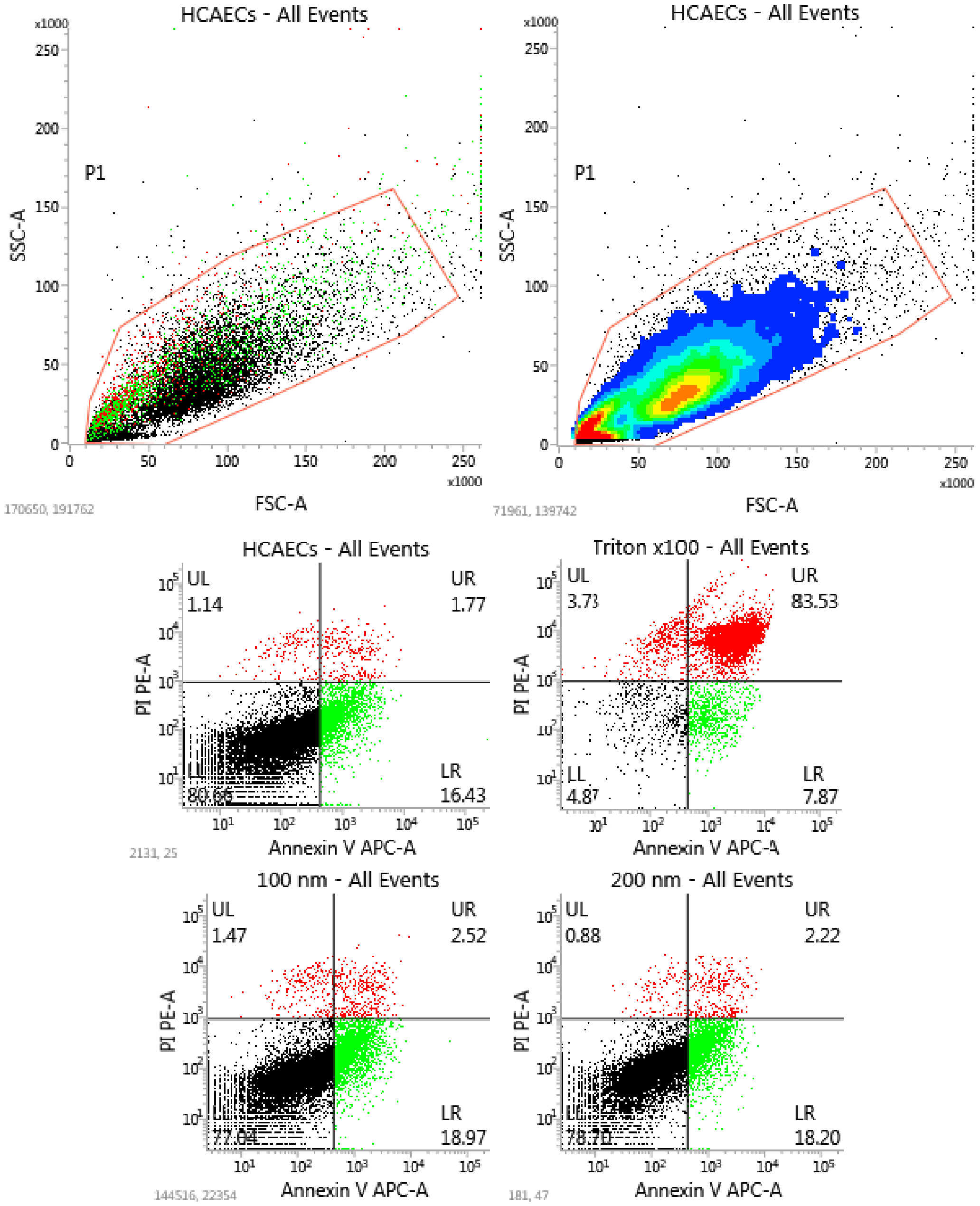


**Figure S1.** An example showing both gated cell population and two-parameter (dual-color fluorescence) scatter density plots obtained from flowcytometry of annexin V and PI double-staining for quantification of apoptotic and necrotic HCAECs cells. The same process was applied for other cell types studied in this work.

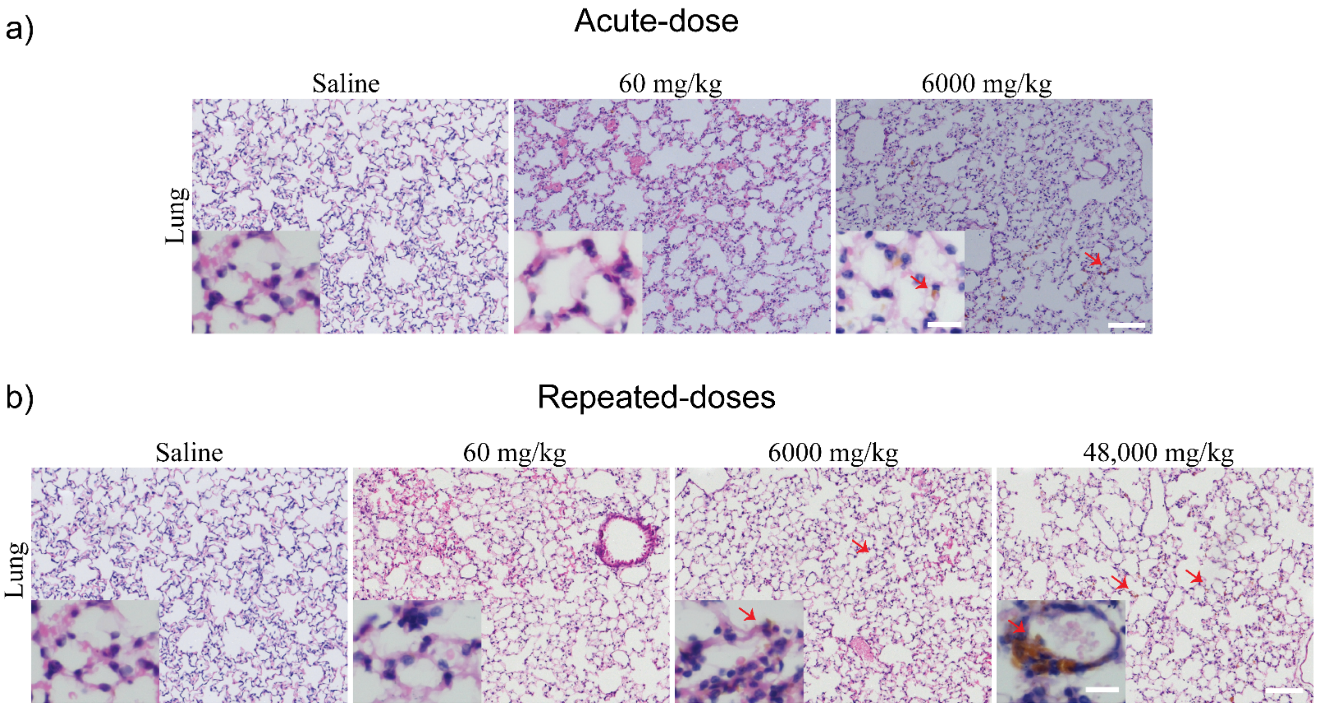


**Figure S2.** Histopathology of mice lungs receiving (**a**) acute and (**b**) accumulated dosages after 14 days following the first IV administration. Sparse PPN deposits (≤ 150 μm) were observed mainly in the liver and spleen at the higher dosages, indicated by red arrows. No major abnormality in lung structure were detected in all PPN concentrations tested. Scale bars are 40 μm (insets) and 100 μm.
